# Associations of Lifestyle Factors with Cadmium and Nickel in Seminal Fluid of Potential Sperm Donors

**DOI:** 10.1007/s12011-026-05015-7

**Published:** 2026-03-10

**Authors:** Dagmar Pilíková, Ondřej Zvěřina, Lucie Burešová, Luděk Fiala, Lucia Zacharová, Jan Šimůnek, Petr Humpolíček

**Affiliations:** 1 Department of Public Health, Faculty of Medicine, Masaryk University, Brno, Czech Republic; 2 Department of Health Care Sciences, Faculty of Humanities, Tomas Bata University in Zlín, Zlín, Czech Republic; 3 Institute of Sexology, First Faculty of Medicine, Charles University, Prague, Czech Republic; 4 Psychiatric Clinic, University Hospital, Faculty of Medicine in Pilsen, Charles University, Pilsen, Czech Republic; 5 Pathological Anatomy Department, Tomas Bata Hospital Zlín, Zlín, Czech Republic; 6 Centre of Polymer Systems, University Institute, Tomas Bata University in Zlín, Zlín, Czech Republic

**Keywords:** Lifestyle factors, Potential sperm donors, Seminal fluid, Cadmium, Nickel, Semen quality

## Abstract

This study aimed to examine the associations between lifestyle factors and concentrations of cadmium (Cd) and nickel (Ni) in seminal fluid (SF) of potential sperm donors, and to determine whether these metals are related to donor qualification status. Between 2015 and 2021, 206 healthy men aged 18–40 were screened as potential sperm donors at reproductive centres in Moravia, Czech Republic. Respondents completed a lifestyle questionnaire covering diet, sedentary behaviour, physical activity, sleep, substance usage, and psychological well-being. Initial semen analysis determined their qualification as donors. Seminal Cd and Ni levels were measured by high-resolution continuum source electrothermal atomic absorption spectrometry (HR-CS ETAAS). Non-parametric methods were used for statistical evaluation. Elevated Cd concentrations were significantly associated with smoking (*P* = 0.008), fish (*P* = 0.024) and confectionery consumption, uninterrupted sedentary time (*P* = 0.017), lower psychological well-being (*P* = 0.024), and sleep duration. Higher fruit (*P* = 0.040) and vegetable (*P* = 0.015) intake resulted in increased seminal Ni concentrations, but without direct association to qualification status. Increased Cd levels were also associated with a bigger size of the settlement (*P* = 0.012). Notably, Cd levels were significantly increased among non-qualified donors (*P* = 0.002), while no such pattern was observed for Ni. No direct significant differences in lifestyle factors were shown between qualified and non-qualified donors. These findings highlight associations between lifestyle factors and seminal Cd levels in potential sperm donors, providing new insights into the role of trace elements in male reproductive health.

**Supplementary Information:**

The online version contains supplementary material available at 10.1007/s12011-026-05015-7.

## Introduction

The use of sperm donors has become a crucial component of assisted reproduction, extending beyond heterosexual couples with male fertility issues to include same-sex female couples and single women [1, 2]. However, sperm donation programs face a persistent challenge in donor recruitment [3], mainly due to poor semen quality among candidates [4–6] and the voluntary withdrawal of the donor at any stage of the donor process [7]. This recruitment problem reflects a broader, well-documented global trend: a consistent decline in semen quality across diverse male populations [8, 9]. The decline in semen quality observed among the male population is frequently linked to detrimental lifestyles [10–14], or with the influence of environmental exposures [15]. Among these environmental exposures, particular attention has been given to toxic metals due to their widespread presence and potential to disrupt male reproductive function. Such exposure can result from air pollution, contamination of both the workplace and the surrounding environment with chemicals (food additives, endocrine disruptors, dioxins, pesticides, herbicides, phthalates, heavy metals) [16, 17], high temperatures [18], and the presence of trace elements [19]. The toxicity of metals can have direct or indirect effects on male reproductive health, acting at the levels of the pre-testicular, testicular, or post-testicular parts of the reproductive axis [20]. Understanding the effects of each metal is, therefore, of great benefit in this context [21, 22]. However, studies describing the effects of individual metals on male reproductive quality have provided highly inconsistent results [22–25]. The present study therefore attempts to clarify the relationships in question.

Within this toxicological framework, Cd and Ni have emerged as metals of particular concern due to their documented effect on the male reproductive system [20, 26]. Cd enters the body mainly from the environment, either by inhalation or through unhealthy lifestyle habits such as smoking [26–28]. Additionally, Cd can be absorbed through the digestive tract, predominantly through contaminated foods, especially cereals, grains, leafy vegetables, potatoes, and offal [29], or *via* the skin [30, 31]. The available literature consistently demonstrates its deleterious effect on sperm quality [17, 25, 32] and the prostate or the vascular system of the testicles [20].

Although previous studies have explored the influence of lifestyle factors [6], such as physical activity [33, 34], and BMI [35] on semen quality in potential sperm donors, none have specifically examined their association with the presence of toxic metals in SF. To our knowledge, this is the first study to investigate the link between modifiable lifestyle behaviours and seminal concentrations of Cd and Ni in potential sperm donors. This fills an important gap in assessing reproductive health and donor qualification, offering new insights for donor screening protocols and male reproductive health education. Therefore, clarification of this relationship offers a novel perspective on male gamete donation.

We hypothesize that lifestyle factors may influence concentrations of Cd and Ni in SF, which may in turn relate to semen quality and potential sperm donor qualification. Understanding these associations will help identify risk factors that impair sperm quality and reduce donor qualification rates. Conversely, mitigating these factors could enhance the likelihood of donor acceptance. Clarifying these associations may not only result in strategies to improve sperm quality but also aligns with broader global health goals. In particular, this research contributes to SDG 3 (Good Health and Well-Being) by supporting preventive strategies and promoting reproductive health literacy among men. The primary aim of this study was to evaluate the association between lifestyle factors and seminal concentrations of Cd and Ni in potential sperm donors. The secondary aims were to compare these associations between qualified and non-qualified donors, and to identify modifiable behaviours that may affect semen quality and thereby inform preventive strategies and reproductive health literacy.

## Materials and Methods

### Study Population

This study was approved by the Ethics Committee of Atlas Hospital JSC (now EUC Clinic Zlín JSC) on 15 May 2015. The study population included 206 potential sperm donors who fulfilled the legal requirements [36–38] for sperm donation in the Czech Republic and voluntarily attended two reproductive centres between 2015 and 2022. All participating centres provided written institutional approval for the use of anonymized donor data. According to Czech legislation, donation is anonymous; donors must be aged between 18 and 40 years and possess at least a secondary school education.

## Data Collection

Data collection consisted of: (1) a self-report **lifestyle questionnaire** covering the previous 3 months, (2) initial **semen analysis** (SPG), and (3) the **chemical analysis** of SF for Cd and Ni. All data were pseudonymized using a unique anonymous code. The lifestyle questionnaire was based on the literature and covered **nutrition**,** sedentary behaviour**,** physical activity**,** sleep**,** addictions**,** and self-reported health including well-being.** It was completed before the initial semen examination. This study included representative variables from the lifestyle domains described above. Variables were selected a priori to represent each domain and to align with the study aims. Additional questionnaire items were analysed but are not presented due to the questionnaire’s breadth and space constraints. Further details can be provided by the corresponding author upon reasonable request.

## Semen Analysis

Semen samples were collected by masturbation in a sterile container and analysed according to the WHO laboratory manual 2010 [39]. To qualify for the donor program, SPG had to meet the following WHO 2010 values: ejaculate volume min 1.5 mL, pH min 7.2, sperm concentration ≥ 15 × 10^6^/mL, total sperm count ≥ 39 × 10^6^/mL, sperm viability 58%, and total sperm motility ≥ 40% (of which progressive ≥ 32%). An aliquot (0.3–0.5 mL) of sample was transferred to a 1 mL Eppendorf-type micro tube and frozen at -20 °C. The samples were later transferred on ice to the laboratory for chemical analysis.

## Determination of Cd and Ni in the Semen Samples

Seminal Cd and Ni concentrations were determined by means of high-resolution continuum source electrothermal atomic absorption spectrometry (HR-CS ETAAS) using a ContrAA 800G spectrometer (Analytik Jena, Germany) after acid digestion of the samples. Approximately 0.5 g of each sample was mixed with 500µL of concentrated nitric acid (Analpure grade, Analytika, Czech Republic) in polypropylene tubes and incubated at 90 °C for four hours in a block heater (Grant QBD2). During digestion, 150µL of hydrogen peroxide (p.a.+ grade, Analytika, Czech Republic) was added to enhance the digestion process. After cooling, the digests were diluted to a final volume of 1 mL with ultrapure water. Samples provided in quantities below 0.4 g were excluded from the study due to insufficient material being available for analysis. To assess method accuracy, procedural blanks, spiked samples, and certified material (Seronorm™ Trace Elements Urine) were processed alongside the samples. The conditions of the measurements and the accuracy parameters are summarized in Supplementary Information I (SI I).

### Statistical Analysis

**Fig. 1:**
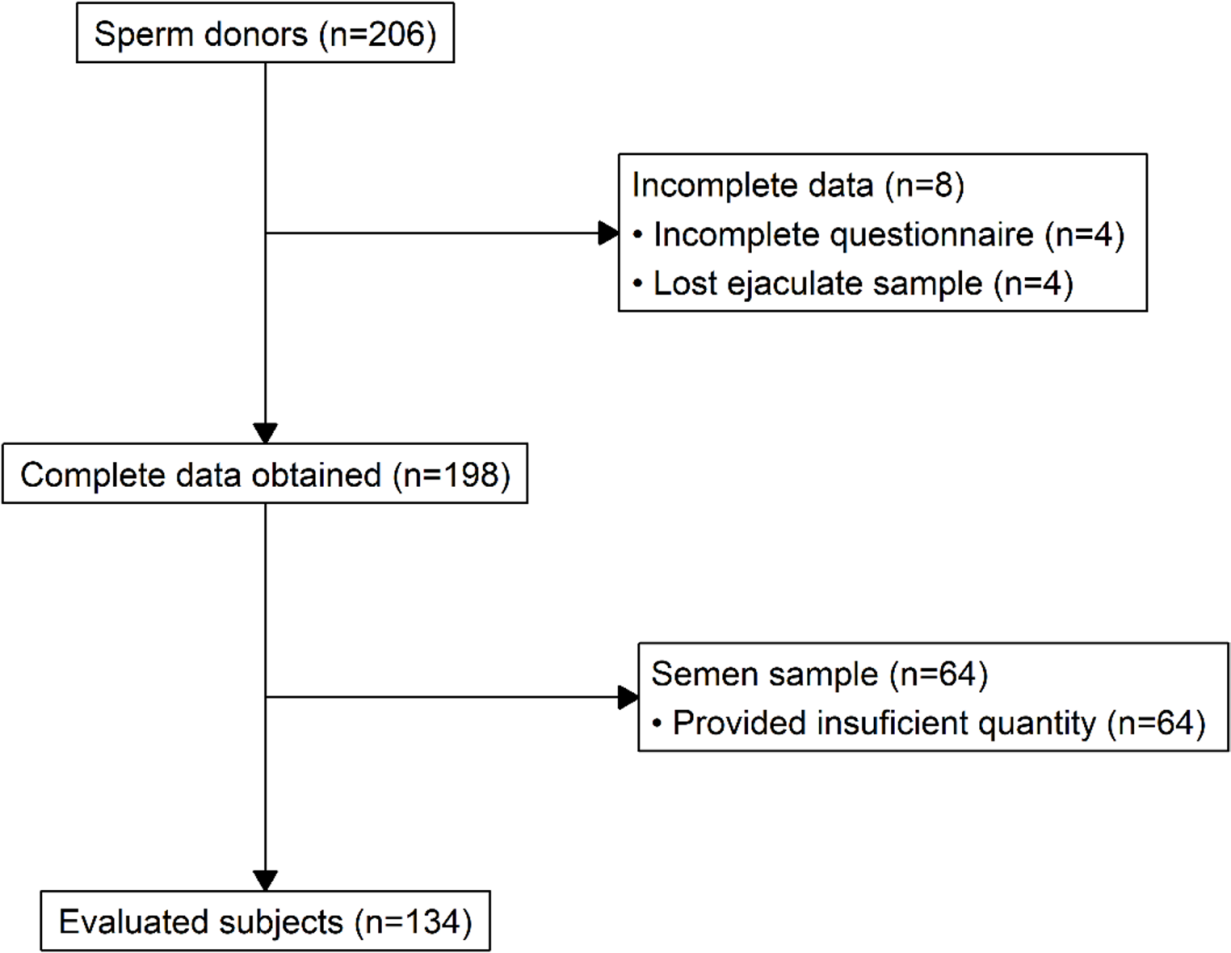
Consort diagram showing respondents’ flow

Of 206 potential sperm donors, only 134 were included in the study (Fig. 1). Descriptive statistics included medians and interquartile range (IQR) for continuous variables, and frequency and percentage for categorical variables. Due to the non-normal distribution of the data (determined by Shapiro-Wilk test), non-parametric methods were used for determination. The Mann-Whitney U test was employed to analyse differences between two groups of continuous variables. Comparison among three or more groups was performed using Kruskal-Wallis analysis of variance followed by Dunn´s post hoc test. Categorical variables were compared using the chi-square test or Fisher’s exact test as applicable. The Spearman correlation coefficient was used to determine the correlation between frequency of food consumption and seminal Cd and Ni concentrations. A P-value of < 0.05 was considered statistically significant. All analyses were performed using R version 4.1.2 [40].

## Results

### Characteristics of the Study Population

The basic characteristics of the potential sperm donors are shown in Table 1. On the basis of the initial semen analysis, 11.2% of the respondents (*n* = 15) qualified for the next stage of the donor program, while 88.8% (*n* = 119) did not meet the required semen quality thresholds. The median age of respondents was 25 years (IQR 21–29). Most had completed high school education (67.2%), and 32.1% held a university degree. At the time of data collection, 55.2% were employed, 28.4% were combining work and study, 15.7% were full-time students, and only 0.7% were unemployed. The median BMI was 24.4 kg/m² (IQR 22.8–26.3).

**Table 1:** Characteristics of the potential sperm donors

Characteristics	Total	Non-qualified	Qualified	
N (%)	134 (100)	119 (88.8)	15 (11.2)	*P*
Age [years]	25.0 [21.0, 29.0]	25.0 [21.0, 28.5]	28.0 [21.5, 30.5]	0.497
Weight [kg]	80.0 [73.3, 90.0]	80.0 [73.0, 90.0]	81.0 [75.5, 100.0]	0.319
High [cm]	182.0 [176.3, 186.0]	182.0 [176.5, 186.0]	182.0 [176.0, 186.5]	0.992
BMI [kg/m2]	24.4 [22.8, 26.3]	24.3 [22.7, 26.0]	26.3 [23.7, 29.5]	0.108
WHO-5 well-being	68.0 [52.0, 76.0]	68.0 [54.0, 78.0]	56.0 [50.0, 70.0]	0.279
Work/study time	40.0 [37.5, 50.0]	40.0 [37.5, 48.0]	50.0 [40.0, 57.5]	0.056
Freq. of Ejaculation	5.0 [4.0, 7.0]	5.5 [4.0, 7.0]	5.0 [4.0, 7.0]	0.751
Education				0.403
High School	90 (67.2)	81 (68.1)	9 (60.0)	
Higher Vocational School	1 (0.7)	1 (0.8)	0 (0.0)	
University 1st dg.	19 (14.2)	18 (15.1)	1 (6.7)	
University 2nd dg.	22 (16.4)	17 (14.3)	5 (33.3)	
University 3rd dg.	2 (1.5)	2 (1.7)	0 (0.0)	
Employment				0.341
Unemployed	1 (0.7)	1 (0.8)	0 (0.0)	
Student	21 (15.7)	20 (16.8)	1 (6.7)	
Student & employment	38 (28.4)	31 (26.1)	7 (46.7)	
Employment (≥ 1)	74 (55.2)	67 (56.3)	7 (46.7)	
Size of the Settlement				**0.012**
< 10 k	43 (32.1)	33 (27.7)	10 (66.7)	
10–50 k	15 (11.2)	13 (10.9)	2 (13.3)	
50–100 k	16 (11.9)	15 (12.6)	1 (6.7)	
> 100 k	60 (44.8)	58 (48.7)	2 (13.3)	
Long-term relationship				0.341
Yes	88 (65.7)	76 (63.9)	12 (80.0)	
No	46 (34.3)	43 (36.1)	3 (20.0)	
Work with Chemicals				0.806
Yes	16 (11.9)	15 (12.6)	1 (6.7)	
No	118 (88.1)	104 (87.4)	14 (93.3)	
BMI category^a^				0.163
Normal	74 (55.2)	68 (57.1)	6 (40.0)	
Overweight	49 (36.6)	43 (36.1)	6 (40.0)	
Obesity	11 (8.2)	8 (6.7)	3 (20.0)	
Dietary supplements				1.000
Use	77 (57.5)	68 (57.1)	9 (60.0)	
Not use	57 (42.5)	51 (42.9)	6 (40.0)	
Medicaments prev. 3 m				0.476
Yes	22 (16.4)	21 (17.6)	1 (6.7)	
No	112 (83.6)	98 (82.4)	14 (93.3)	

Data show Median [IQR interquartile range] or frequency (percentage)

The bold values show significant results at a significance level of *P* < 0.05

^a^BMI was categorised by WHO criteria [42] and zero respondents were underweight

The majority of respondents (65.7%) reported being in a long-term relationship. The average number of ejaculations per week was five, with no significant difference between the qualified and unqualified groups (*P* = 0.751). The vast majority (88.1%) of respondents reported no daily exposure to harmful substances such as toxic chemicals, radiation, or biological agents. Likewise, 83.6% had not taken any medication in the previous 3 months. Regarding place of the settlement, 44.8% lived in cities with > 100,000 inhabitants, while 32.1% resided in areas with fewer than 10,000. A statistically significant difference in donor qualification was observed in relation to size of the settlement (*P* = 0.012).

### Influence of Demographic Factors

The concentrations of seminal Cd and Ni were analysed in relation to key demographic factors such as age, education level, employment status, relationship status, and size of the settlement (Table 2). No significant associations were observed for age, education, or employment. In age-adjusted models, Cd concentration showed no significant correlation with age (r_S_ = 0. 084, *P* = 0.332) (SI II). However, significant differences in seminal Cd concentrations according to size of the settlement (*P* = 0.020) were observed. Respondents residing in towns with fewer than 10,000 inhabitants exhibited the lowest seminal Cd concentrations (0.074 µg/L, IQR 0.060–0.098), while those from cities with over 100,000 inhabitants had the highest levels (0.103 µg/L, IQR 0.073–0.141). Moreover, a statistically significant association was observed between Cd levels and relationship status (*P* = 0.004). No comparable association was found for Ni levels (Table 2).

**Table 2:** Differences between demographic factors and concentration of cd and Ni in SF

Characteristics		Cd [µg/L]		Ni [mg/L]	
	*N* (%)	Median [IQR]		Median [IQR]	
Total	134 (100)	0.090 [0.067, 0.126]	*P*	0.040 [0.033, 0.044]	*P*
Size of the Settlement			**0.020**		0.741
< 10k	43 (32.1)	0.074 [0.060, 0.098]		0.040 [0.036, 0.044]	
10k–50k	15 (11.2)	0.098 [0.067, 0.149]		0.043 [0.034, 0.044]	
50k–100k	16 (11.9)	0.102 [0.076, 0.126]		0.037 [0.029, 0.043]	
> 100k	60 (44.8)	0.103 [0.073, 0.141]		0.039 [0.033, 0.045]	
Age			0.252		0.631
18–20 year	24 (17.9)	0.088 [0.070, 0.130]		0.040 [0.032, 0.044]	
21–25 year	51 (38.1)	0.081 [0.057, 0.108]		0.040 [0.035, 0.044]	
26–30 year	39 (29.1)	0.102 [0.075, 0.135]		0.038 [0.033, 0.045]	
> 30 years	20 (14.9)	0.092 [0.067, 0.122]		0.037 [0.031, 0.043]	
Long-term Relationship			**0.004**		0.550
Yes	88 (65.7)	0.081 [0.059, 0.116]		0.040 [0.034, 0.044]	
No	46 (34.3)	0.105 [0.079, 0.147]		0.038 [0.033, 0.043]	

Data show Median [IQR interquartile range] or frequency (percentage)

The bold values show results at a significance level of *P* < 0.05

**Fig. 2:**
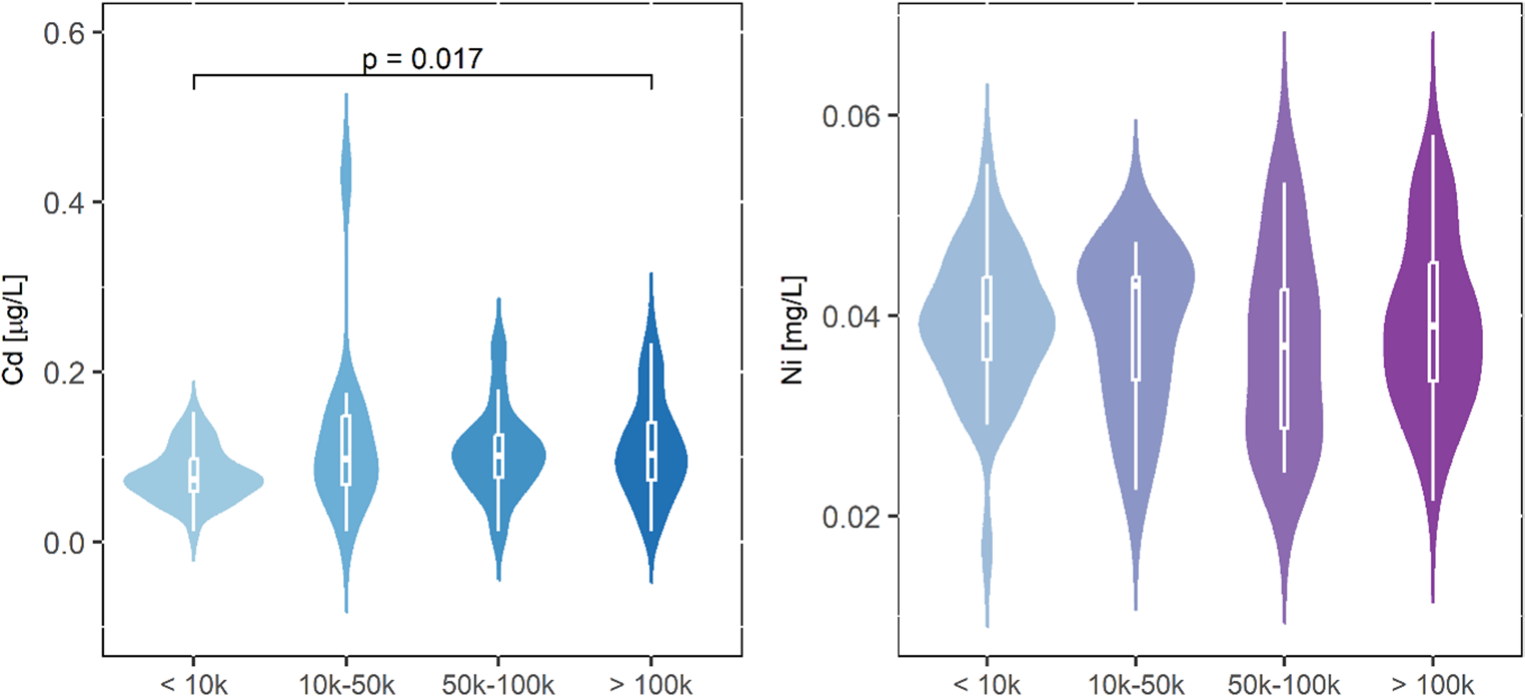
Differences in seminal Cd and Ni concentrations across settlement size categories. The statistical difference was observed between Cd levels of volunteers from cities with less than 10,000 inhabitants and less th0 inhabitantsan

Additional Dunn’s post hoc tests revealed statistically significant differences in seminal Cd concentrations between settlement size categories. Specifically, respondents residing in cities with over 100,000 inhabitants had significantly higher Cd levels compared to those from towns with fewer than 10,000 inhabitants (*P* = 0.017; Fig. 2). The pairwise comparison between these two groups showed a median difference of 0.029 µg/L (0.103 µg/L vs. 0.074 µg/L). No other pairwise comparisons between the remaining settlement size categories reached statistical significance. (*P* = 0.017; Fig. 2).

### Lifestyle Habits and Donor Qualification

**Fig. 3:**
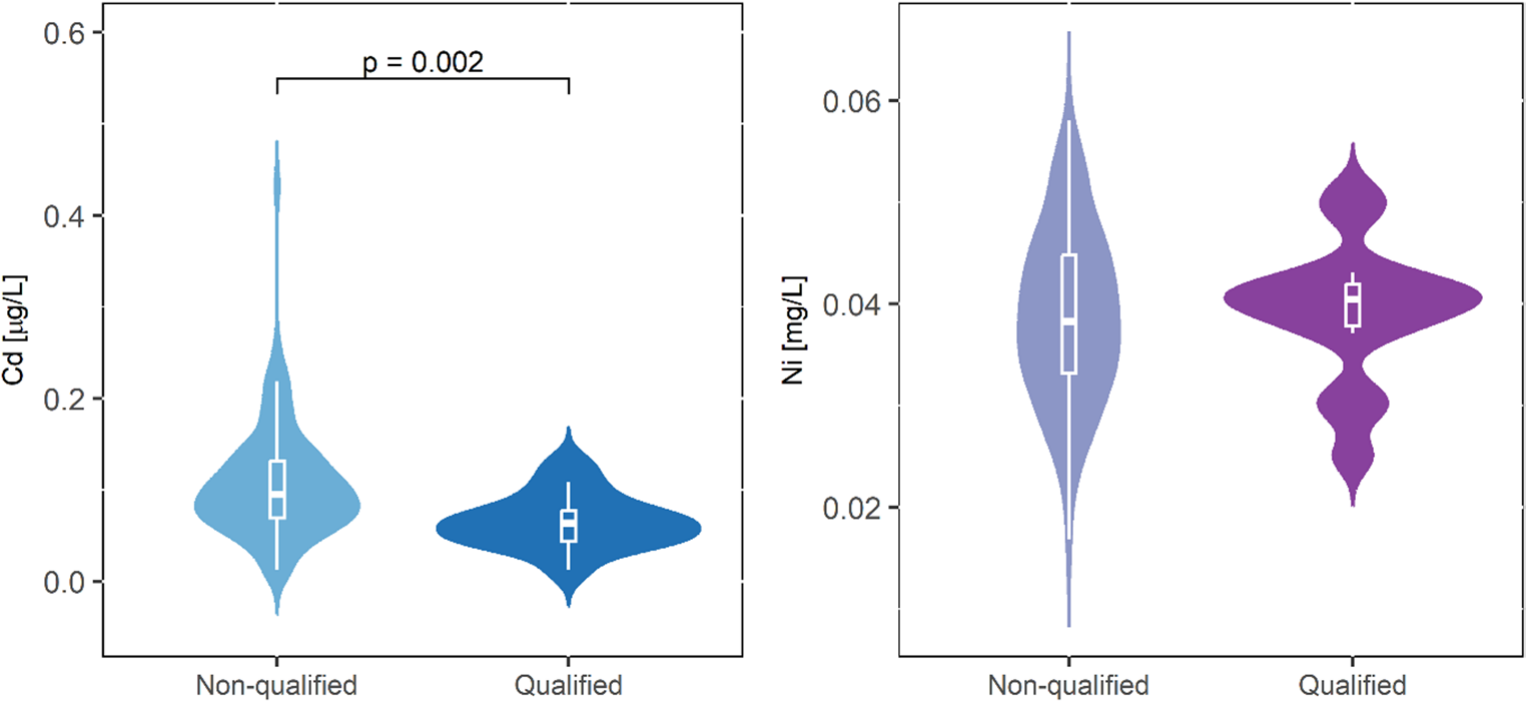
Seminal Cd and Ni levels in relation to donor eligibility

No significant association was found between lifestyle factors and qualification for the donor program (Table 3). These results suggests that lifestyle factors alone did not predict eligibility in this cohort. In contrast, a strong significant association was observed between seminal Cd concentrations and donor qualification status. Qualified donors showed markedly lower Cd levels (median 0.064 µg/L; IQR 0.044–0.077) compared to unqualified donors (median 0.095 µg/L; IQR 0.069–0.132), with a P-value of 0.002 (Fig. 3, SI III). No such difference was observed for seminal Ni concentrations (*P* = 0.714).

**Table 3:** Associations between lifestyle habits and donor qualification

Characteristics	Total	Non-qualified	Qualified	
Total N (%)	134 (100)	119 (88.8)	15 (11.2)	*P*
Sport				0.216
None	7 (5.2)	7 (5.9)	0 (0.0)	
Fitness	37 (27.6)	35 (29.4)	2 (13.3)	
Cycling	20 (14.9)	19 (16.0)	1 (6.7)	
Other	70 (52.2)	58 (48.7)	12 (80.0)	
Sedentary				0.937
< 2 h	31 (23.1)	27 (22.7)	4 (26.7)	
2–6 h	72 (53.7)	64 (53.8)	8 (53.3)	
> 6 h	31 (23.1)	28 (23.5)	3 (20.0)	
Uninterrupted sedentary				0.351
< 1 h	70 (52.2)	64 (53.8)	6 (40.0)	
1–2 h	49 (36.6)	41 (34.5)	8 (53.3)	
> 2 h	15 (11.2)	14 (11.8)	1 (6.7)	
Smoking^*^				0.561
Non-smoker	85 (63.4)	76 (63.9)	9 (60.0)	
Ex-smoker	22 (16.4)	18 (15.1)	4 (26.7)	
Occasional	18 (13.4)	16 (13.4)	2 (13.3)	
Smoker	9 (6.7)	9 (7.6)	0 (0.0)	
Alcohol per week				0.200
0 g	30 (22.4)	29 (24.4)	1 (6.7)	
1–20 g	91 (67.9)	77 (64.7)	14 (93.3)	
21–39 g	10 (7.5)	10 (8.4)	0 (0.0)	
≥ 40 g	3 (2.2)	3 (2.5)	0 (0.0)	
WHO-5 well-being				0.805
≤ 50	28 (20.9)	24 (20.2)	4 (26.7)	
>50	106 (79.1)	95 (79.8)	11.73.3)	
Sleeping I				1.000
Regular	115 (85.8)	102 (85.7)	13 (86.7)	
Irregular	19 (14.2)	17 (14.3)	2 (13.3)	
Sleeping II				1.000
≥ 7 h	72 (53.7)	64 (53.8)	8 (53.3)	
< 7 h	62 (46.3)	55 (46.2)	7 (46.7)	

Data show Median [IQR interquartile range] or frequency (percentage). Significance level of P value < 0.05

^*^Non-smoker = never smoked; Ex-smoker = previously smoked but has not smoked for >3 months; Occasional smoker = smokes <1 cigarette/day; Smoker = smokes ≥1 cigarette/day and reports long-term smoking

These results highlight Cd as a potential environmental factor influencing semen quality and eligibility for sperm donation.

### Association between Lifestyle Habits and Cd and Ni Concentrations

#### Dietary Habits

Dietary intake was assessed using the food frequency questionnaire (FFQ) [41] expanded to 21 items, reflecting dietary patterns over the previous 3 months. The original 18-food item questionnaire was supplemented by 3 further food items (soy, nuts, and processed and instant foods). The majority of respondents (90.3%) reported no adherence to dietary restrictions. Among the remainder, 9.7% reported being vegetarian or reducing their intake of fat, sugar, or meat. Three subjects reported occasional fasting, and one described a fitness-oriented diet. Moreover, the BMI, classified by WHO criteria [42], revealed that 55.2% of the participants were of **normal weight**, 36.6% were **overweight**, 8.2% were **obese**, and none of the respondents were underweight (Table 1). No significant associations were found between BMI categories or dietary restrictions and the seminal concentrations of Cd (*P* = 0.697 and *P* = 0.668, respectively) and Ni (*P* = 0.642 and *P* = 0.991, respectively) in SF (Table 4).

**Table 4:** Differences in cd and Ni concentrations in SF between lifestyle variables

Characteristics		Cd [µg/L]		Ni [mg/L]	
	*N* (%)	Median [IQR]		Median [IQR]	
Total	134 (100)	0.090 [0.067, 0.126]	*P*	0.040 [0.033, 0.044]	*P*
BMI category^a^			0.697		0.642
Normal BMI	74 (55.2)	0.089 [0.065, 0.118]		0.038 [0.034, 0.043]	
Overweight	49 (36.6)	0.101 [0.068, 0.133]		0.040 [0.032, 0.046]	
Obesity	11 (8.2)	0.082 [0.063, 0.115]		0.037 [0.033, 0.042]	
Water source			0.101		0.753
Tap water	102 (76.1)	0.089 [0.068, 0.131]		0.039 [0.033, 0.045]	
Bottled	27 (20.1)	0.095 [0.067, 0.118]		0.040 [0.033, 0.044]	
Other	5 (3.7)	0.053 [0.044, 0.071]		0.035 [0.034, 0.041]	
Dietary restriction			0.668		0.991
No restriction	121 (90.3)	0.092 [0.068, 0.125]		0.040 [0.033, 0.044]	
Restriction	13 (9.7)	0.085 [0.053, 0.150]		0.037 [0.034, 0.042]	
Daily water intake			0.380		0.441
≥ 1.5 L	113 (84.3)	0.088 [0.066, 0.125]		0.040 [0.033, 0.044]	
< 1.5 L	21 (15.7)	0.107 [0.076, 0.132]		0.037 [0.034, 0.041]	
Daily position (work/school time)			0.874		0.381
Standing & mostly standing	42 (31.3)	0.093 [0.067, 0.126]		0.039 [0.036, 0.045]	
Mostly sitting	34 (25.4)	0.086 [0.053, 0.126]		0.039 [0.030, 0.042]	
Sitting	58 (43.3)	0.090 [0.068, 0.121]		0.040 [0.033, 0.047]	
Sedentary			0.939		0.883
< 2 h	31 (23.1)	0.095 [0.065, 0.132]		0.040 [0.036, 0.043]	
2–6 h	72 (53.7)	0.086 [0.066, 0.126]		0.039 [0.033, 0.045]	
> 6 h	31 (23.1)	0.088 [0.068, 0.112]		0.040 [0.033, 0.043]	
Uninterrupted sedentary			**0.017**		0.804
< 1 h	70 (52.2)	0.095 [0.069, 0.126]		0.040 [0.034, 0.045]	
1–2 h	49 (36.6)	0.073 [0.057, 0.108]		0.040 [0.033, 0.043]	
> 2 h	15 (11.2)	0.108 [0.098, 0.177]		0.038 [0.034, 0.044]	
IPAQ-short			0.833		0.409
Vigorous	35 (26.1)	0.080 [0.066, 0.120]		0.040 [0.036, 0.045]	
Moderate	8 (6.0)	0.092 [0.038, 0.125]		0.032 [0.028, 0.044]	
Low	91 (67.9)	0.094 [0.067, 0.126]		0.038 [0.033, 0.044]	
Sport activity			0.943		0.709
None	7 (5.2)	0.090 [0.071, 0.115]		0.040 [0.036, 0.043]	
Fitness	37 (27.6)	0.095 [0.065, 0.138]		0.038 [0.034, 0.043]	
Cycling	20 (14.9)	0.106 [0.066, 0.132]		0.036 [0.032, 0.042]	
Other	70 (52.2)	0.088 [0.068, 0.118]		0.040 [0.033, 0.045]	
Smoking			**0.008**		0.678
Non-smoker	85 (63.4)	0.094 [0.069, 0.125]		0.040 [0.033, 0.043]	
Ex-smoker	22 (16.4)	0.119 [0.087, 0.150]		0.039 [0.033, 0.043]	
Occasional	18 (13.4)	0.063 [0.048, 0.089]		0.041 [0.034, 0.048]	
Smoker	9 (6.7)	0.081 [0.067, 0.141]		0.038 [0.034, 0.040]	
Alcohol			0.715		0.395
0 g	30 (22.4)	0.084 [0.067, 0.135]		0.037 [0.033, 0.043]	
1–20 g	91 (67.9)	0.090 [0.066, 0.126]		0.040 [0.033, 0.044]	
21–39 g	10 (7.5)	0.090 [0.070, 0.099]		0.041 [0.034, 0.047]	
≥ 40 g	3 (2.2)	0.126 [0.096, 0.180]		0.045 [0.041, 0.050]	
Marijuana			0.592		0.379
Total	131^*^ (100.0)	0.090 [0.067, 0.126]		0.040 [0.033, 0.044]	
Zero intake	58 (44.3)	0.095 [0.068, 0.136]		0.037 [0.033, 0.043]	
Intake	73 (55.7)	0.089 [0.060, 0.118]		0.040 [0.034, 0.045]	
CD^c^ or surgery in history			0.162		0.183
No	118 (88.1)	0.088 [0.065, 0.121]		0.040 [0.033, 0.045]	
Yes	16 (11.9)	0.099 [0.071, 0.176]		0.036 [0.032, 0.041]	
Medication prev. 3 months			0.794		0.173
Yes	23 (17.2)	0.098 [0.061, 0.126]		0.035 [0.029, 0.044]	
No	111 (82.8)	0.090 [0.068, 0.121]		0.040 [0.034, 0.044]	
Type of ejaculation			0.291		0.694
By masturbation	87 (64.9)	0.097 [0.067, 0.133]		0.040 [0.033, 0.045]	
By coitus	47 (35.1)	0.082 [0.066, 0.114]		0.038 [0.033, 0.043]	
Ejaculation frequency (times/per wk)			0.292		0.072
≤ 5	68 (50.7)	0.095 [0.071, 0.129]		0.040 [0.034, 0.045]	
> 5	66 (49.3)	0.085 [0.062, 0.118]		0.037 [0.031, 0.043]	
Sleeping I		0.112		0.801	
Regular	115 (85.8)	0.092 [0.068, 0.130]		0.040 [0.033, 0.044]	
Irregular	19 (14.2)	0.075 [0.048, 0.108]		0.040 [0.034, 0.044]	
Sleeping II			**0.039**		0.138
≥ 7 h	72 (53.7)	0.095 [0.071, 0.134]		0.040 [0.034, 0.046]	
< 7 h	69 (46.3)	0.083 [0.055, 0.114]		0.037 [0.033, 0.043]	
WHO-5 well-being			0.244		0.700
≤ 50	28 (20.9)	0.105 [0.071, 0.135]		0.040 [0.035, 0.045]	
> 50	106 (79.1)	0.088 [0.065, 0.118]		0.039 [0.033, 0.044]	
Well-being by Likert^b^			**0.024**		0.513
Very good	30 (22.4)	0.099 [0.080, 0.140]		0.037 [0.031, 0.043]	
Good	69 (51.5)	0.076 [0.060, 0.109]		0.038 [0.033, 0.043]	
Neutral	29 (21.6)	0.103 [0.067, 0.132]		0.040 [0.036, 0.044]	
Poor	6 (4.5)	0.139 [0.108, 0.170]		0.044 [0.035, 0.047]	

Bold values show significant results from statistical analysis at a significance level of P< 0.05

^a^BMI was categorised by WHO criteria [42] and zero respondents were underweight

^b^Zero respondents recorded “very poor” mental well-being

^c^Chronical Disease

^*^Three respondents refused to answer

Although overall dietary patterns showed no direct association with donor eligibility (see SI IV, V) or seminal Cd and Ni, the analysis of individual food items revealed specific links. The Spearman correlation coefficient confirmed a statistical dependence between Cd concentrations and the consumption of confectionery (sweets/candy) and fish, while the consumption of fruit and vegetables correlated with the concentration of Ni in SF. The subsequent Mann-Whitney tests confirmed the statistical significance of these associations (Table 5).

These findings suggest that specific dietary components, rather than broad patterns of body composition, may influence seminal exposure to the examined metals.

**Table 5:** Mann-Whitney analysis results for the median of the consumption of foodstuffs and cd and Ni in SF

Foodstuffs		Cd [µg/L]		Foodstuffs		Ni [mg/L]	
	*N* (%)	median [IQR]			*N* (%)	median [IQR]	
Total	134 (100)	0.090 [0.067, 0.126]	*P*	Total	134 (100)	0.040 [0.033, 0.044]	*P*
Sweets/candy			**0.025**	**Fruit**			**0.040**
≤ 0.214	73 (54.5)	0.101 [0.076, 0.132]		≤ 0.786	83 (61.9)	0.038 [0.032, 0.043]	
> 0.214	61 (45.5)	0.075 [0.053, 0.118]		> 0.786	51 (38.1)	0.040 [0.035, 0.046]	
Fish			**0.024**	**Vegetable**			**0.015**
≤ 0.214	120 (89.6)	0.088 [0.065, 0.122]		≤ 0.786	83 (61.9)	0.038 [0.031, 0.042]	
> 0.214	14 (10.4)	0.116 [0.088, 0.167]		> 0.786	51 (38.1)	0.041 [0.035, 0.046]	
Red meat			0.842	**Red meat**			0.163
≤ 0.5	109 (81.3)	0.089 [0.068, 0.125]		≤ 0.5	109 (81.3)	0.040 [0.034, 0.044]	
> 0.5	25 (18.7)	0.101 [0.054, 0.129]		> 0.5	25 (18.7)	0.037 [0.029, 0.043]	

Average daily frequency coefficients: 0.214 represents the consumption of food 1–2 times per wk; 0.5 represents 3–4 times per wk, and 0.786 represent 5–6 times per wk

Bold values show significant results from the statistical analysis at a significance level of *P* < 0.05

Average daily frequency coefficients: 0.214 represents the consumption of food 1–2 times per wk; 0.5 represents 3–4 times per wk, and 0.786 represent 5–6 times per wk.

Regarding water intake, 84.3% of respondents adhered to the WHO recommended minimum of 1.5 L per day. No significant association was observed between water intake and the seminal concentrations of Cd (*P* = 0.380) and Ni (*P* = 0.441). The most frequently-reported sources of drinking water were as follows: **tap water** (76.1%); **bottled water** (20.1%); and **other** sources of water (e.g. filtered) (3.7%). Similarly, no significant association between water source and metal concentrations in SF was observed (Table 4).

#### Sedentary Behaviour and Physical Activity

**Fig. 4:**
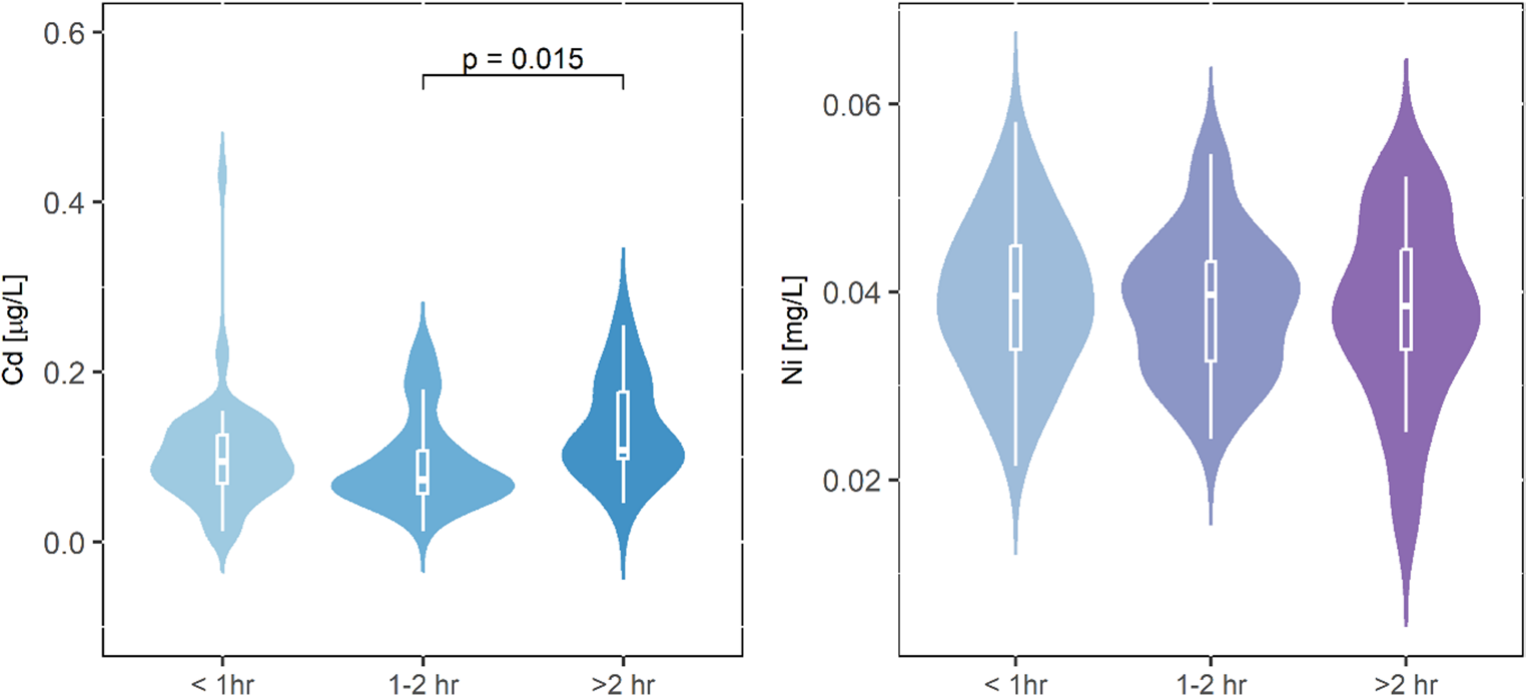
Seminal metal concentrations across uninterrupted sedentary time categories

The **sedentary behaviour** and physical activity of potential sperm donors were evaluated using self-reported measures (Table 4). The respondents reported their typical daily posture (sitting, mostly sitting, standing, or mostly standing), and whether they spent more than two hours sitting without a break [43, 44]. While the majority of respondents (43.3%) reported primarily sitting during the day, no significant association was observed with seminal concentrations of Cd or Ni. However, prolonged uninterrupted sitting was significantly associated with increased seminal Cd concentration (*P* = 0.017). Post hoc Dunn’s test revealed a statistically-significant increase in seminal Cd concentration in donors reporting sitting for more than 2 h compared to those sitting for 1–2 h (*P* = 0.015; Fig. 4).

On the other hand, regarding physical activity, 94.8% of respondents reported participation in some form of sport. Fitness (27.6%) and cycling (14.2%) were the most common. Physical activity was assessed using the short form of the Intensity of Physical Activity Questionnaire (IPAQ-short), and classified under low, moderate, or vigorous intensity according to MET scores. Neither the intensity nor type of specific sport showed a statistically significant relationship with seminal Cd or Ni concentrations. Notably, cycling was associated with the highest median of seminal Cd levels, but this difference did not reach statistical significance.

#### Substance use – smoking, alcohol, Illicit Drugs

**Fig. 5:**
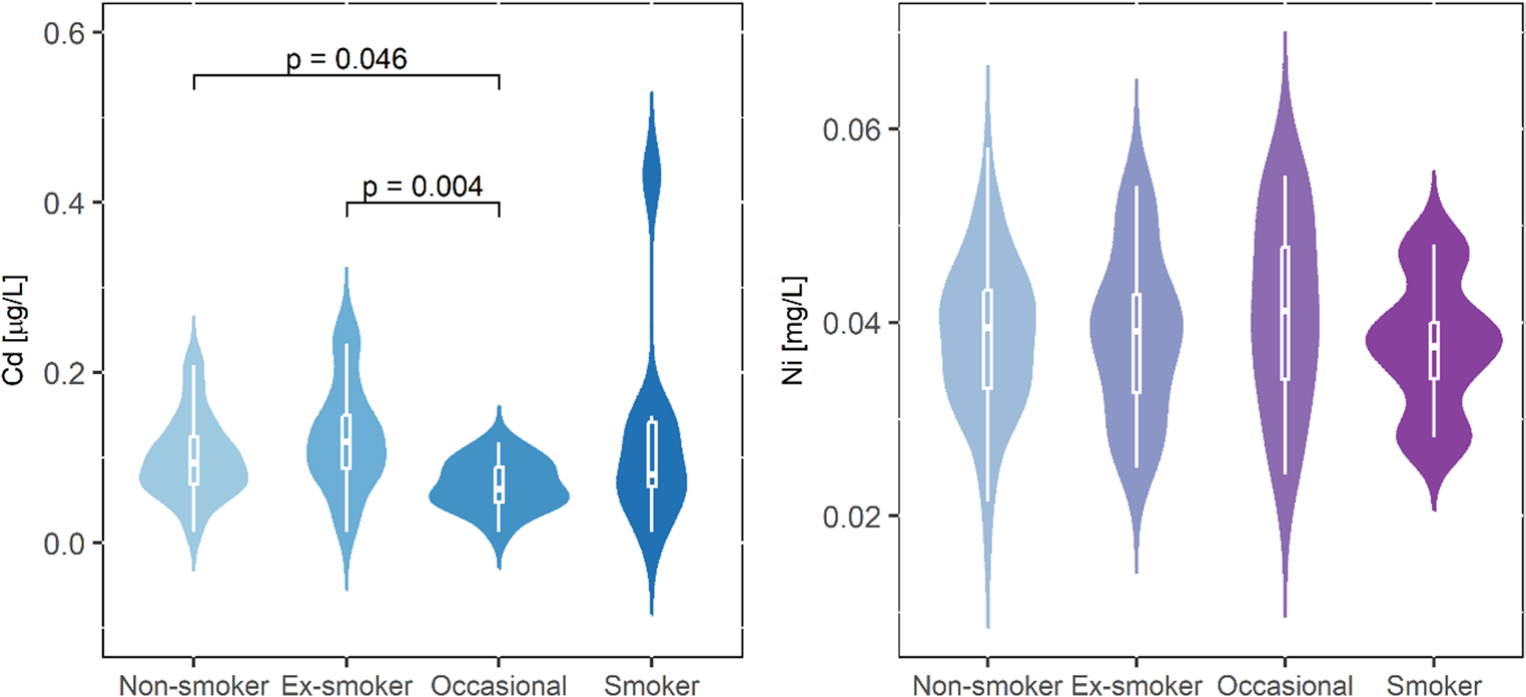
Seminal Cd and Ni concentrations by smoking status

Smoking, alcohol consumption, and the use of illicit drugs were self-reported by respondents (Table 4). The majority were non-smokers (63.4%), while 16.4% identified themselves as ex–smokers, 13.4% as occasional smokers, and 6.7% as regular smokers. A significant association was found between smoking status and seminal Cd concentration (*P* = 0.008). The highest median Cd level was measured in the group of ex-smokers (0.119 µg/L, IQR 0.087–0.150). No significant relationship was found with levels of Ni (*P* = 0.678). Post hoc Dunn’s test revealed a statistically-significant higher Cd concentration in the group of ex-smokers compared to occasional smokers, and in the group of occasional smokers compared to non-smokers (Fig. 5).

Alcohol consumption was categorised into four groups (zero intake, 1–20 g/day, 21–39 g/day, ≥ 40 g/day). No significant association was identified between alcohol consumption and the measured seminal concentrations of Cd (*P* = 0.715) and Ni (*P* = 0.395).

In addition, the use of illicit drugs was also recorded in these categories: marijuana, dance drugs, cocaine, heroin, methamphetamine, LSD, and solubilizers. Three individuals chose to refrain from responding to this question. Marijuana had been tried by 55.7% of respondents, and 9.2% reported using it within the last 3 months. However, one respondent (0.8%) confirmed regular marijuana use over the previous 3 months. The use of other substances, including dance drugs (10.7%), LSD or similar hallucinogens (8.4%), and cocaine or heroin (6.1%), was rare and not regular. No statistical association was observed between illicit drug use and Cd or Ni concentration in SF.

#### Sleeping and well-being

Sleeping habits and mental well-being were assessed as examples of general health indicators. According to WHO recommendations, adult males should sleep a minimum of seven hours per night. In this study, 46.3% of respondents reported sleeping less than seven hours per night. In the group that met the WHO sleep recommendation, the chemical analysis yielded a significantly greater seminal Cd concentration (0.095 µg/L, IQR 0.071–0.134) compared to those who did not (0.083 µg/L, IQR 0.055–0.114) with a p-value at 0.039. Meanwhile, no significant relationship was observed for seminal Ni levels.

Mental health was evaluated using two scales: the WHO-5 well-being index and a 5-point Likert scale. While WHO-5 did not reveal a significant association with seminal metal concentrations, the Likert scale indicated a statistically-significant link between Cd levels and perceived well-being (*P* = 0.024). Increased Cd concentrations were measured in potential sperm donors reporting neutral or poor well-being. No relationship was found with the concentration of Ni in SF.

In addition to the aforementioned lifestyle habits, data pertaining to basic information on general health including sexual behaviour, surgical history and medication use were also recorded. The analysis did not reveal any significant relationship with either seminal Cd or Ni – Table 4.

These findings highlight that, while most general lifestyle factors—including diet, physical activity, and alcohol use—were not significantly associated with seminal Cd and Ni concentrations, selected behaviours and exposures demonstrated relevant links. Increased Cd concentrations were observed among donors who reported prolonged uninterrupted sitting, past smoking, and poorer mental well-being. Additionally, specific dietary components such as fish and confectionery intake showed a significant association with Cd levels. In contrast, Ni concentrations remained stable across most examined parameters and showed no consistent associations, except a significant association with fruit and vegetable consumption. The results highlight potentially modifiable factors that may contribute towards influencing Cd concentrations in the SF of potential sperm donors.

## Discussion

This study provides novel insights into the potential role of lifestyle-related factors in influencing seminal Cd and Ni concentrations in potential sperm donors. Studies examining lifestyle-metal relationships in the context of donor qualification remain scarce. Evidence from donor-screening cohorts supports the relevance of trace element exposure for semen quality. In a cohort of 1428 healthy men screened as potential sperm donors, Chen et al. [45], reported associations between urinary essential elements and semen quality and highlighted potential windows of susceptibility. Complementing this approach, Liu et al. [46] quantified multiple exogenous metals directly within spermatozoa at single-cell resolution in men screened as sperm donors and showed that metal heterogeneity and prevalence were associated with semen quality parameters.

### Linking Donor Qualification with Seminal Cd and Ni Concentration

Semen quality is essential for the qualification of a potential donor into the donor program. The most prominent finding of this study was the significantly higher Cd concentration in SF of unqualified potential sperm donors. These results reinforce the suspected detrimental effect of Cd on semen quality [47, 48], which is consistent with previous studies linking elevated Cd levels with impaired sperm motility [49–51], sperm morphology and reactive oxygen specious (ROS) [32, 52, 53], low sperm viability [25], and hormonal synthesis [26, 54]. However, some studies have reported no significant impacts of Cd on semen parameters [55, 56], highlighting ongoing debate in the field.

Although toxic metals have been extensively studied in infertile populations, only a few studies have focused on potential sperm donors [22]. Our findings align with the partial results of Benoff et al. [55], who observed the lowest levels of Cd in the seminal fluid of sperm donors with normal semen parameters when compared to infertile men and the general population (statistically significant data). The observed median Cd concentrations in our qualified cohort were comparable to theirs, despite differences in the qualification criteria and study populations. In comparison to the unqualified cohort, a Finnish study [57] comparing sperm donor candidates with workers in a refinery and polyolefin factory found higher seminal Cd levels in the donor group, despite there being no significant differences in semen quality. These results suggest that even individuals undergoing donor screening may carry environmental burdens of cadmium exposure. In addition, conference data Chen et al. [58] have suggested intra-individual variability of cadmium in seminal plasma, which should be considered when interpreting single-sample measurements in donor-screening settings.

Cadmium exerts its toxic effects on male reproduction through both direct and indirect mechanisms. Its ability to mimic essential ions such as calcium and zinc disrupts cellular homeostasis and enzymatic systems, while its strong affinity for sulfur-containing compounds interferes with protein structure and function [54]. In the male reproductive organs, cadmium accumulates particularly in the epididymis, seminal vesicles and prostate, where it induces excessive generation of ROS, leading to oxidative stress, the apoptosis of germ cells, and degeneration of seminiferous tubules [28, 48]. Direct effects include the impaired viability and endocrine function of Leydig and Sertoli cells, as well as reduced sperm motility, morphology, and fertilization capacity [32, 49, 50, 52]. Indirect pathways involve endothelial dysfunction, inflammation, disruption of the hypothalamic–pituitary–gonadal axis, and endocrine-disrupting effects manifested as impaired steroidogenesis and testosterone imbalance [26, 54]. Collectively, these processes compromise spermatogenesis, endocrine regulation, and ultimately male reproduction health.

A significant association was also found between seminal Cd concentrations and size of the settlement. Potential donors living in larger cities (> 100,000 inhabitants) showed higher seminal Cd levels than those from smaller towns. This supports the findings by De Franciscis et al. [27], who reported higher levels in more industrialized areas. Conversely, Olszak-Wasik et al. [59] observed unexpectedly higher seminal Cd concentrations among men in agricultural regions, suggesting regional variation in exposure sources. The results of these studies point to different sources of Cd in different regions across the world. As already mentioned, Cd can enter the body both from the air and from contaminated food. Thus, from an epidemiological point of view, it is necessary to monitor Cd concentrations not only in the air but also in the soil or surface water of the environment, as they can adversely affect male reproductive health through the Cd they contain. The measurement of Cd levels in SF of potential sperm donors from non-contaminated, routinely inhabited areas is rarely studied. Therefore, the study provides unique data on the content of metals in the SF of potential sperm donors from this part of the country. Taken together, these findings suggest that measuring Cd concentrations in seminal fluid, though not commonly part of donor assessment, could offer additional insights into environmental risk factors influencing donor eligibility.

In contrast, the seminal Ni concentration showed no significant association with donor qualification in our study, although its accumulation may impose toxicity in the testis [20]. This study aligns with previous reports suggesting that seminal Ni levels may not directly affect sperm quality between cohorts of men with good or pathological SPG [19]. However, due to the limited and heterogeneous data available in this context, the potential role of Ni remains uncertain and warrants further investigation.

It is important to note that donor qualification criteria vary considerably across countries and reproductive centres, which may limit the direct comparability of qualification rates and semen quality thresholds in different studies [3, 6, 7, 60, 61].

### Linking Lifestyle Factors with Seminal Cd and Ni Concentration

Several lifestyle-related variables showed statistically-significant associations with seminal Cd concentrations. In this section, we discuss the most relevant factors: smoking, diet, sedentary behaviour, sleep, and mental well-being, in light of existing literature.

#### Smoking

In this study, smoking was significantly associated with higher seminal Cd concentrations (*P* = 0.008), a finding consistent with established literature identifying tobacco smoke as a major non-occupation source of Cd exposure [28, 62, 63]. Notably, the highest Cd levels were observed in ex-smokers, which supports the hypothesis of cadmium bioaccumulation and its prolonged retention in body tissues – even after the cessation of exposure [64]. This supports the claim by Bernard at al. [65, 66] about the cumulative properties of this metal. The range of its biological half-life is from 10 to as much as 40 years [67–70] in various tissues, including liver, lungs, spleen, pancreas, heart and testes [26, 66, 68, 70]. Its presence in the male reproductive tract, particularly the testes and accessory glands, has been linked to altered semen quality [69]. Although Cd has a long biological half-life and age-related bioaccumulation might be expected, additional analyses in this cohort showed no significant correlation between age and seminal Cd. This lack of correlation is plausibly due to the narrow donor age range and the dominant influence of modifiable exposures (diet, smoking) over cumulative age-related load. These findings indicate that, in this cohort of potential sperm donors, age played only a minor role in seminal Cd accumulation, while smoking was a significant determinant. While the prevalence of current smokers in our cohort was low (6.7%), the total prevalence of male smokers in the Czech Republic is much higher [71]. This may reflect either the selective recruitment of healthier donors or potential underreporting by donors due to the social stigma associated with smoking in the context of gamete donation. Regardless, our findings suggest that even occasional or ex-smokers can leave a measurable Cd footprint in the male reproductive system.

In contrast, smoking was not a significant factor with respect to Ni concentrations in SF in this study. This supports the findings by Rodríguez-Díaz et al. [72], who found no significant relationship with smoking status and Ni levels.

#### Dietary Habits

For individuals not occupationally exposed to toxic metals and who do not smoke, diet represents the primary source of Cd and Ni intake [30, 68]. In our study, specific dietary patterns showed significant associations with seminal metal levels, despite overall dietary restrictions and BMI not influencing Cd or Ni concentrations.

Fish consumption more than 1–2 times per week was associated with increased seminal Cd concentrations. This frequency aligns with the WHO’s recommendations for healthy fish intake, suggesting that even standard dietary habits may contribute to measurable Cd exposure. Cd content in fish has been demonstrated by studies analysing fish from various European and African regions, where Cd levels occasionally exceeded permissible thresholds [73–76]. Although most commercial products remain within tolerable limits [73], cumulative intake remains a concern, particularly for individuals with higher fish consumption. Fish are considered a minor source of dietary Cd intake [76]. The tolerable weekly intake of cadmium per kg of body weight was explored by CONTAM in 2011 and was established to be 2.5 µg/kg of body weight [77]. The absorption and toxic effects of Cd from food can be prevented by a balanced diet rich in vitamins, polyphenols and antioxidants [69].

In contrast, fruit and vegetable intake was significantly associated with seminal Ni concentrations, even at frequencies below WHO recommendations (median consumption was 5–6 times per week). Food monitoring studies from Poland and Libya confirm the presence of Ni in common produce, though typically within tolerable daily intake limits [78, 79]. The highest levels of Ni were found in spinach, romaine lettuce, mango, and bananas, depending on the region and agricultural practices. While these EFSA TDI limits may not represent immediate systemic toxicity, our findings suggest a relevance for reproductive health even at relatively low levels of exposure. On the other hand, studies from Nigeria measuring Ni levels in watermelon [80], pineapple and banana [81] present worrying results showing that Ni contents exceed WHO permissible limits.

These results highlight the fact that the contents of metals contained in foodstuffs depend on numerous environmental factors including soil composition, water quality, and the regional agricultural practices where the foodstuffs come from. Although current EFSA guidelines and WHO recommendations provide safety thresholds, even with regard to reproductive toxicology, it is mentioned only marginally [82]. Despite increasing recognition of the toxicity of certain metals to reproduction, semen remains an underexplored biological matrix in regulatory toxicology. The EFSA does not currently consider seminal Cd or Ni levels in its reproductive health assessments. Our findings contribute to emerging evidence suggesting that even low-level dietary exposure may manifest itself in seminal fluid, this highlighting a potential gap in current reproductive risk monitoring frameworks.

#### Prolonged Sitting

Sedentary behaviour is an emerging factor of concern in reproductive health research. According to WHO guidelines, prolonged sitting is associated with increased risks of cardiovascular diseases, cancer, and type-2 diabetes, as well as with decreased mental well-being [83, 84]. Moreover, in the context of male fertility, sedentary time has been linked to increased scrotal skin temperature (SST) [85, 86]. Prolonged sitting has been shown to increase SST [43, 44, 87], which in turn may impair spermatogenesis through oxidative stress and DNA damage, and reduce sperm motility [88–90]. In contrast, Sun et al. [33] did not confirm any adverse effects of sedentary behaviour on sperm parameters.

This study revealed a statistically-significant association between uninterrupted sitting ≥ 2 h and elevated seminal Cd concentrations. To the best of our knowledge, this is the first study to report a relationship between sedentary time and seminal Cd levels. This finding raises the possibility that sedentary habits may facilitate Cd accumulation in the male reproductive tract or amplify its local effects through increased heat stress.

Although, the mechanism remains unclear and the relationship between sedentary habits and Cd content in SF may not represent a direct causal link, it is plausible that prolonged sitting alters local circulation or thermoregulation in a way that modulates metal retention or toxicity. As a modifiable lifestyle factor, sedentary time may represent a promising target for health education and primary prevention efforts aimed at, and optimizing male reproductive health. Its reduction could, in turn, decrease the concentration of Cd in SF and thus contribute to improving the semen quality of potential sperm donors. Future studies should further investigate the potential synergistic effect of toxic metals and environmental heat stress on spermatogenesis.

#### Sleeping and well-being

Emerging evidence suggests that exposure to certain metals, particularly Cd, may negatively affect neurobehavioral health, including sleep regulation and psychological well-being [91–93]. However, the interplay between metal concentration in SF and these lifestyle variables remains largely unexplored.

The present study described a statistically-significant association between seminal Cd concentration and self-reported sleep duration. Interestingly, elevated seminal Cd levels were observed in potential sperm donors following the recommended sleep duration of at least 7 h/day, which contradicts earlier findings that linked elevated blood Cd levels with reduced sleep duration or increased risk of sleep disorders [91, 92]. This discrepancy may be attributed to differences in biological matrices (blood vs. seminal plasma), population characteristics, or the underlying exposure routes. Although the mechanism remains unclear, this result may suggest the presence of either indirect links or confounding factors affecting both sleep habits and Cd bioaccumulation. Future research using objective sleep quality measures (e.g., actigraphy or polysomnography) and detailed environmental exposure data is needed to validate these findings.

Similarly, the present study revealed a significant association between increased seminal Cd concentration and poorer mental well-being. Specifically, individuals experiencing a worse mood on a 5-point Likert scale exhibited elevated seminal Cd levels, and this trend was also observed for lower well-being indices (≤ 50) using the WHO-5 well-being index, although the results were not statistically significant. These findings align with prior studies associating higher Cd levels in blood or urine with depressive symptoms in general American adult populations [94, 95].

Several biological mechanisms may underline these associations. Cd exposure has been shown to induce oxidative stress, disrupt endocrine function [48], and exert neurotoxic effects [92], all of which may impact both reproductive and mental health. However, due to the paucity of studies in this area specifically addressing seminal Cd levels and psychological outcomes, caution is warranted in interpreting these results. Further investigation into the bidirectional relationship between mental well-being and reproductive health in the male population, including potential sperm donors, is strongly recommended.

In this cohort, no significant association was found between levels of seminal Ni and either sleep duration or well-being indicators.

These results support the relevance of addressing modifiable lifestyle factors in reproductive health counselling. The novelty of this research lies in its integration of lifestyle assessment, semen analysis, and trace metal quantification within a single cohort of potential sperm donors. These findings emphasize the importance of considering under-recognized sources of Cd exposure—i.e., those other than smoking—in the context of male reproductive health. Moreover, the findings support the incorporation of targeted health education and preventive strategies into donor recruitment and broader reproductive health promotion, including SDG 3 (target 3.7), which emphasizes health literacy and proactive care in male reproductive health.

### Limitation

The current study has, however, several limitations that should be considered when interpreting the findings. First, it focused on a specific cohort of potential sperm donors undergoing standard screening procedures, without detailed andrological evaluation. Therefore, the findings cannot be directly generalized to the wider male population. Second, lifestyle data were obtained through a self-reported questionnaire, which may be subject to recall or social desirability bias. Nevertheless, the anonymous nature of the participation likely reduced the risk of intentional misreporting. Third, the study relied on the reproductive centres’ final decisions regarding donor qualification. While this reflects real-world clinical practice, it limits the assessment of semen quality. Finally, due to the cross-sectional design of the research, causal relationships between lifestyle factors, concentrations of metals, and donor qualification cannot be established. Despite these limitations, the fact that the cohort in the present study was relatively large and well-characterized strengthens the relevance and applicability of the findings within the context of male reproductive health.

## Conclusion

This study identifies statistically-significant associations between selected lifestyle factors and seminal Cd and Ni concentrations in the SF of potential sperm donors. Elevated seminal Cd levels were associated with smoking, fish consumption, uninterrupted sitting, poorer mental well-being, and, unexpectedly, recommended sleep duration. Seminal Ni levels were linked to fruit and vegetable intake. Additionally, significant differences in seminal Cd concentration were observed according to the size of the settlement, suggesting the potential role of environmental exposure.

Importantly, the study highlights that elevated seminal Cd concentrations were associated with a lower likelihood of donor qualification, whereas no such association was found for Ni or for lifestyle factors alone. These findings underscore the importance of considering environmental toxicants in the context of male fertility screening.

The study provides novel insights into the interplay between lifestyle habits, environmental exposure, and reproductive health in a well-defined male donor population. Its results support the relevance of health education and risk awareness among potential sperm donors and may inform future strategies to improve donor recruitment and fertility preservation. However, further research is needed to confirm these associations and explore potential causal pathways.

## Supplementary Information

Below is the link to the electronic supplementary material.

Supplementary Material 1 (PDF 522 KB)

## Data Availability

Data that support the findings of this study are available from the corresponding author upon reasonable request.

## Abbreviations

Cd: cadmium
CONTAM: the Panel of Contaminants in the Food Chain
EFSA: European Food Safety Authority
IQR: interquartile range
Ni: nickel
SDG: Sustainable Development Goals
SF: seminal fluid
SPG: spermiogram
SST: Scrotal Skin Temperature
TDI: Tolerable Daily Intake
WHO: World Health Organization
