# Supplementary material for: Associations of Lifestyle Factors with Cadmium and Nickel in Seminal Fluid of Potential Sperm Donors
Source: Biol Trace Elem Res. 2026 Mar 10;204(7):5045–61. doi: 10.1007/s12011-026-05015-7 (PMC13319362; doi:10.1007/s12011-026-05015-7)
Supplement: Supplementary file 1 — Supplementary Material 1 (PDF 522 KB) [file 12011_2026_5015_MOESM1_ESM.pdf]

Associations of Lifestyle Factors with Cadmium and Nickel in Seminal Fluid of Potential Sperm Donors

Biological Trace Element Research

Supplementary Data I Operating conditions for the determination of cadmium and nickel in samples using HR-CS ETAAS and the analytical characteristics of the method

**Parameter**	**Cd**	**Ni**
wavelength (nm), pixels used	228.802 nm (central pixel ±1)	232.003 nm (central pixel ± 1)
pyrolysis temperature	400 °C	1,100 °C
atomization temperature	1,500 °C	2,325 °C
injected sample volume (ETAAS)	20 µL	20 µL
modifier	permanent coating W/Ir	5 µL $Mg(NO_3)_2$
LOD (sample)[1]	0.008 µg/L	0.12 µg/L
LOQ (sample)[1]	0.025 µg/L	0.4 µg/L
spike recovery[2]	96–106 %	98–107 %
CRM Seronorm™ Urine (certified)	4.6 ± 0.4 µg/L	50.4 ± 3.2 µg/L
CRM Seronorm™ Urine (found, bias)[3]	4.7 ± 0.4 µg/L (+2%)	53 ± 5 µg/L (+ 5%)

[1] Detection (LOD) and quantification limit (LOQ) are calculated as three/ten times the standard deviation, respectively, of ten measurements of a blank solution, taking into the account the dilution of the samples during digestion.
[2] Recovery of the analyte from the spiked samples (n = 3).
[3] Trueness expressed as a bias (percentual difference between certified and obtained value).

Supplementary Data II Correlation between age and Cd concentration in SF of potential sperm donors

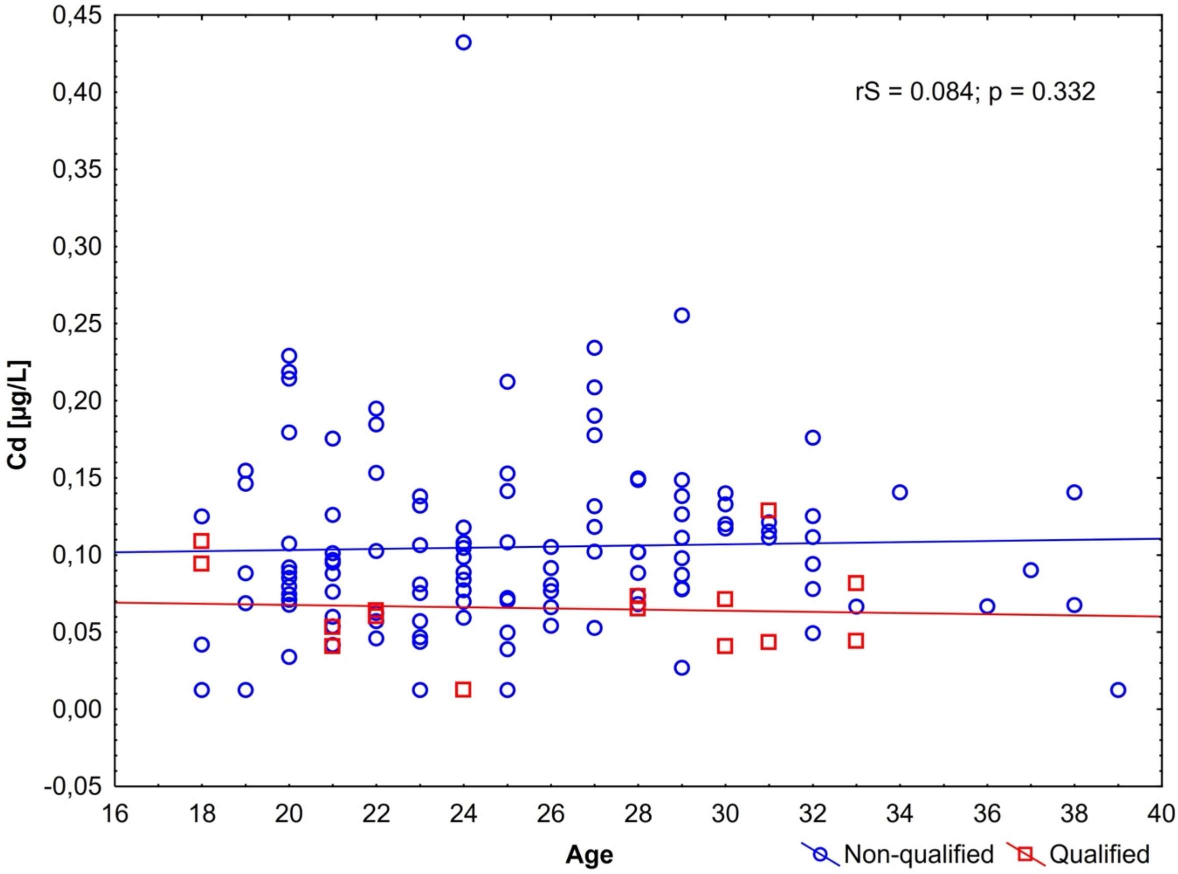

Supplementary Data III Associations between Cd and Ni concentrations in SF and the eligibility of potential sperm donors

**Heavy metals**	**Total**	**Non-qualified**	**Qualified**	
N (%)	134 (100)	119 (88.8)	15 (11.2)	*P*
Cd [µg/L] (median [IQR])	0.090 [0.067, 0.126]	0.095 [0.069, 0.132]	0.064 [0.044, 0.077]	**0.002**
Ni [mg/L] (median [IQR])	0.040 [0.033, 0.044]	0.038 [0.033, 0.045]	0.040 [0.038, 0.042]	0.714

Data show Median values [IQR interquartile range].
Bold values show significant results from the statistical analysis.

Supplementary Data IV Association between food pyramid* and the eligibility of potential sperm donors

**Characteristics**	**Total**	**Non-qualified**	**Qualified**	
N (%)	134 (100)	119 (88.8)	15 (11.2)	*P*
Number of meals per day	4.10 [3.50, 4.80]	4.20 [3.50, 4.80]	4.00 [3.60, 4.30]	0.534
Cereals	2.14 [1.86, 2.79]	2.14 [1.86, 2.86]	2.43 [2.00, 2.61]	0.608
Vegetables	1.21 [1.00, 1.50]	1.21 [1.00, 1.50]	1.21 [0.71, 1.39]	0.949
Fruits	0.79 [0.50, 1.00]	0.79 [0.50, 1.00]	0.79 [0.64, 1.00]	0.753
Diary products	0.79 [0.50, 1.00]	0.79 [0.50, 1.00]	0.79 [0.50, 1.00]	0.705
Meat, fish, eggs. legumes	2.36 [1.93, 3.21]	2.36 [1.93, 3.21]	2.86 [2.21, 3.18]	0.321
Top of the pyramid	0.50 [0.43, 1.00]	0.50 [0.43, 1.00]	0.50 [0.43, 0.86]	0.685

Data show Median [IQR interquartile range]. P values are at a significance level < 0.05.
* Based on Food Frequency Questionnaire by Fiala [41]

Supplementary Data V Spearman's correlation coefficient (colour-coded) and corresponding p-values (numeric values) between seminal Cd and Ni concentrations in foodstuffs of FFQ*

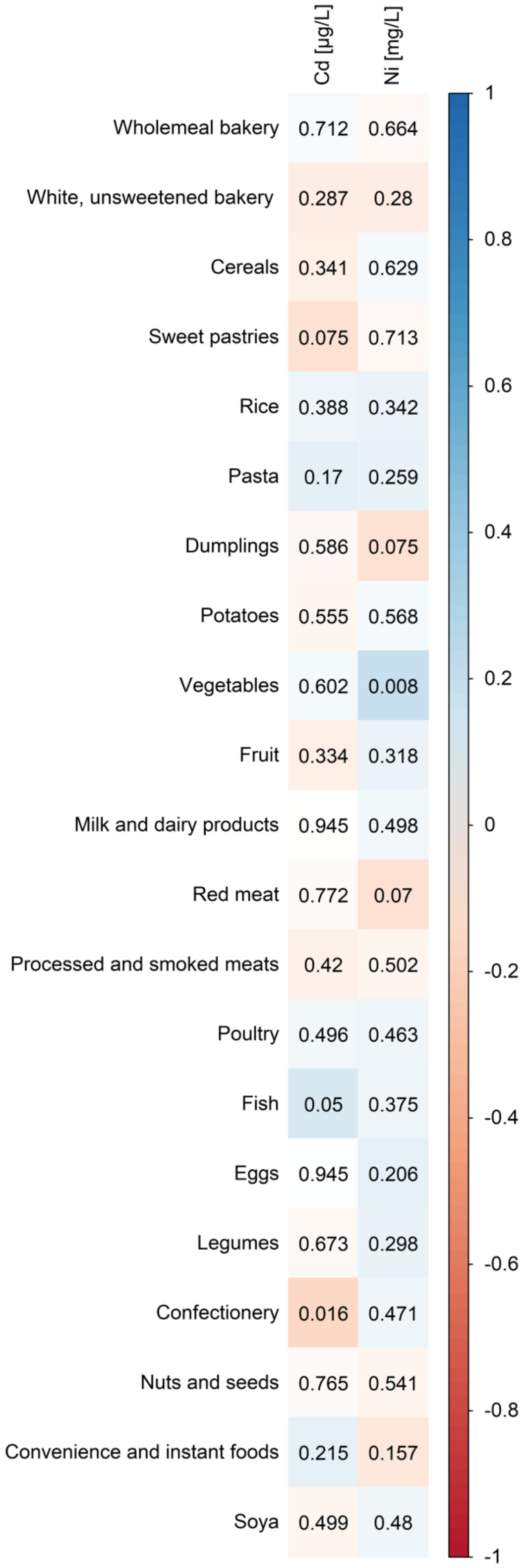

note: * Based on Food Frequency Questionnaire by Fiala [41]
